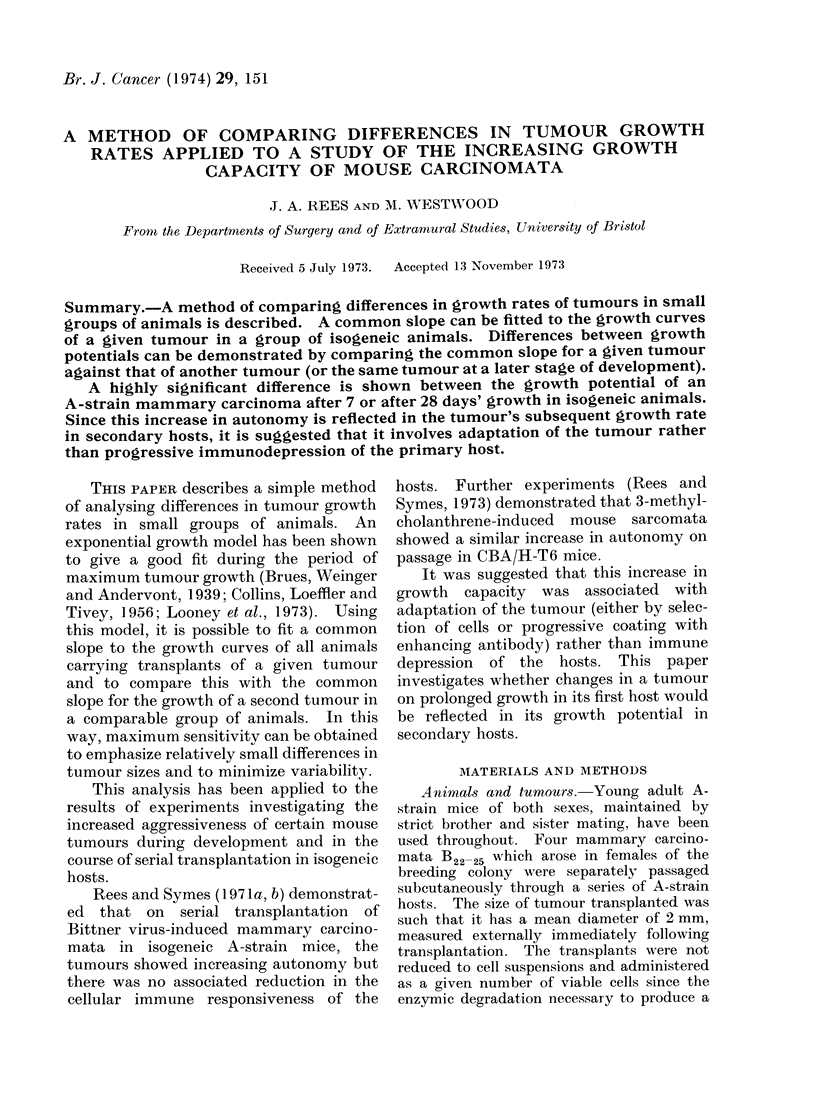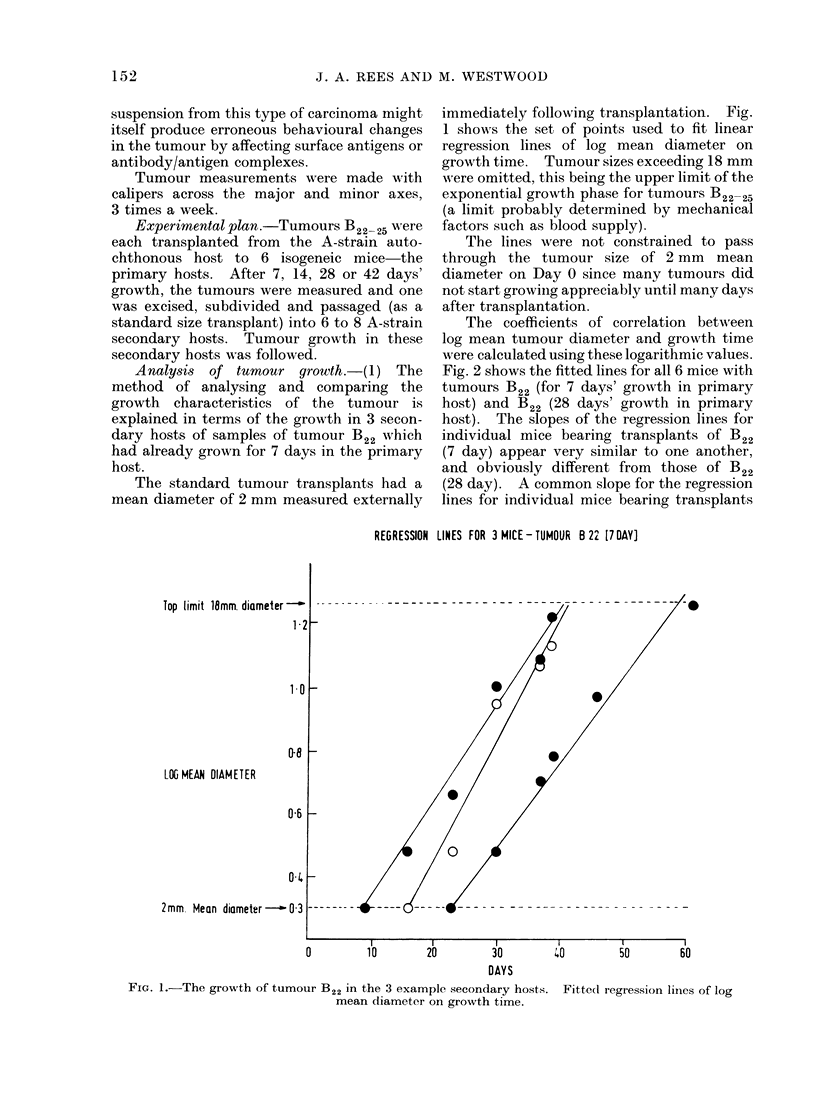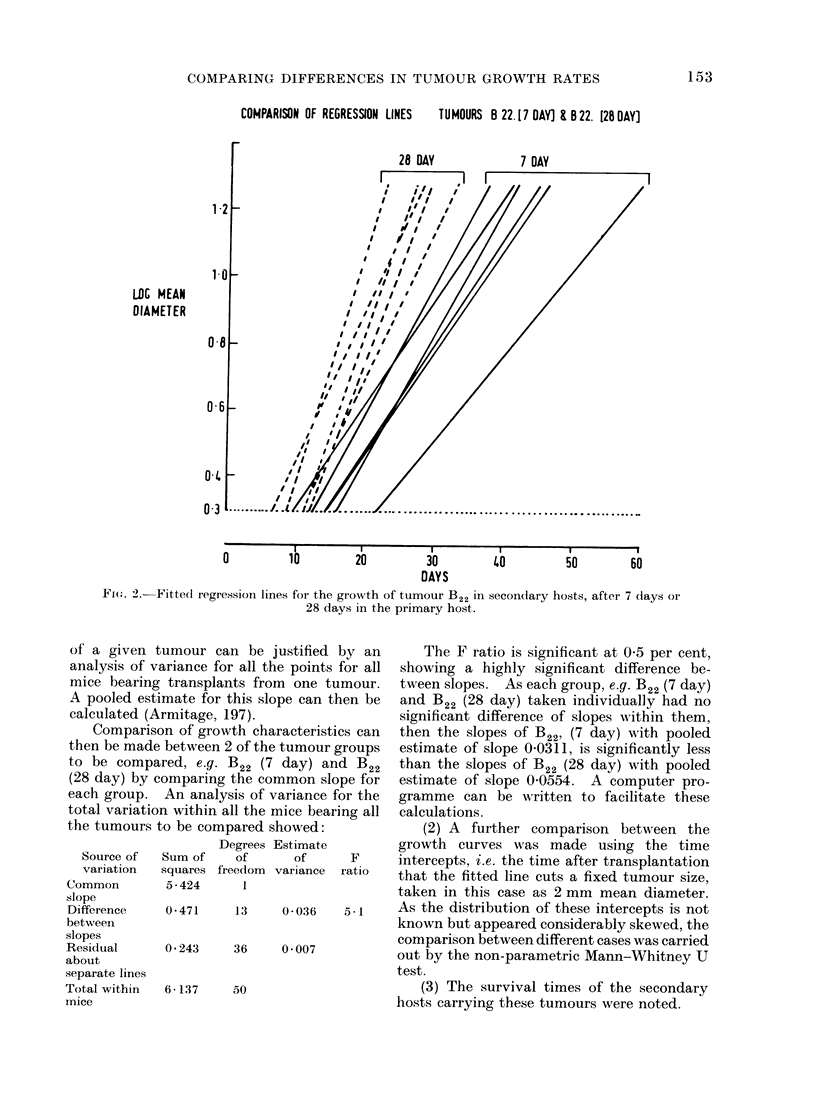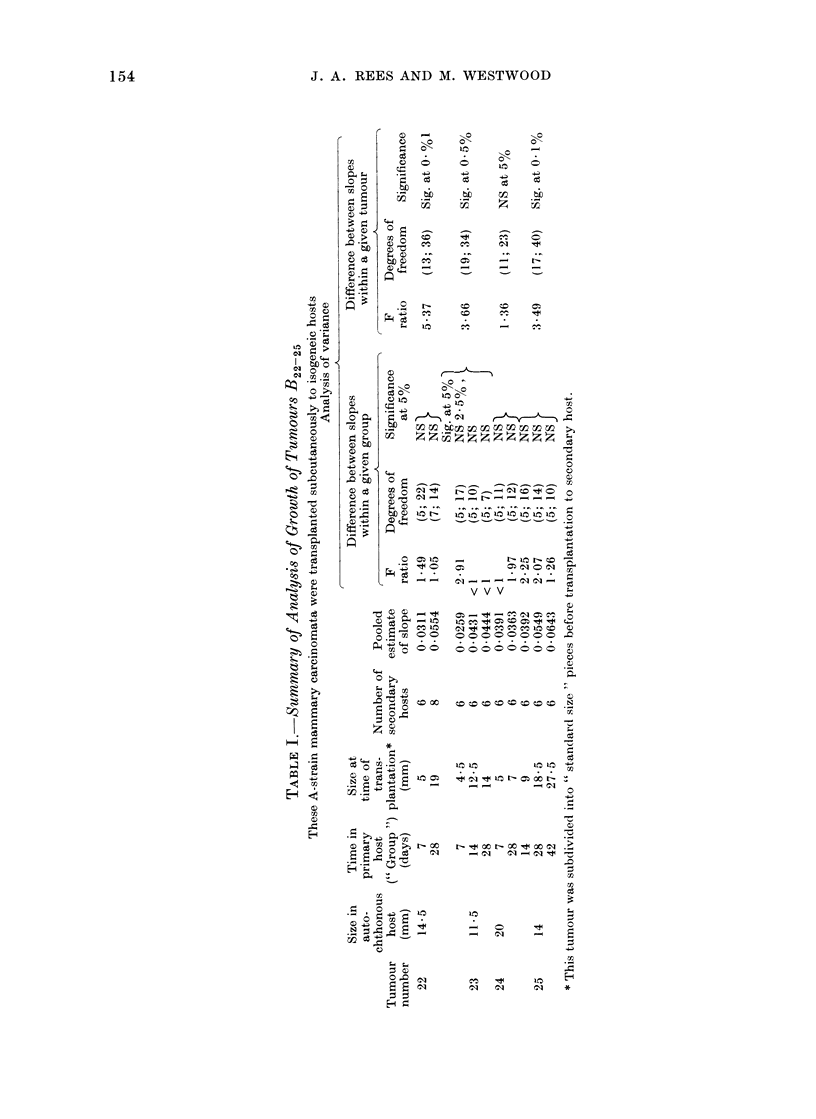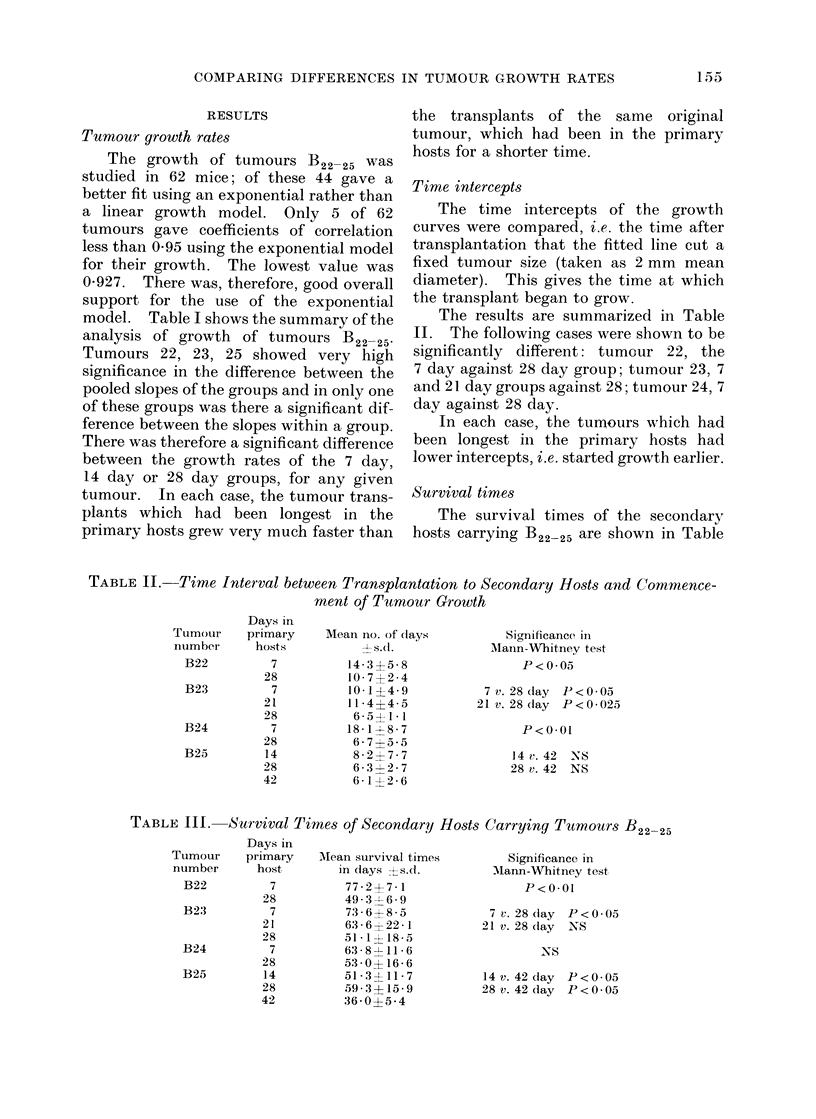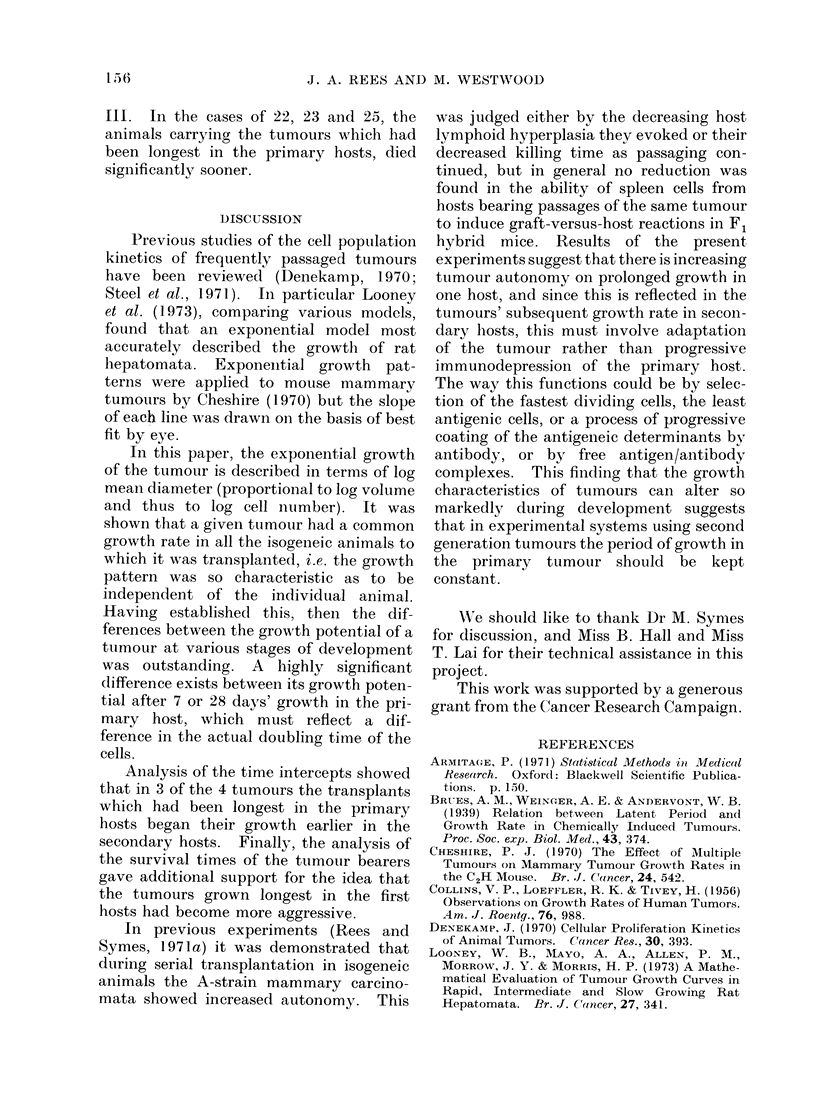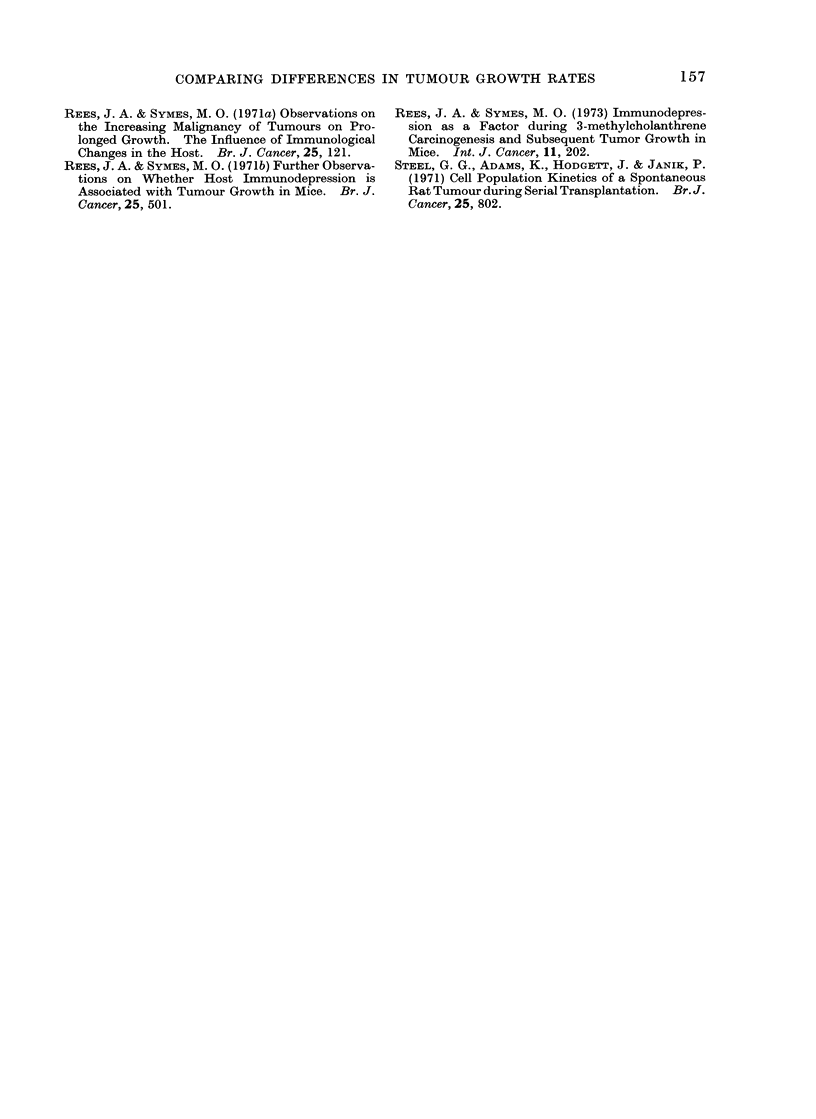# A Method of Comparing Differences in Tumour Growth Rates Applied to a Study of the Increasing Growth Capacity of Mouse Carcinomata

**DOI:** 10.1038/bjc.1974.51

**Published:** 1974-02

**Authors:** J. A. Rees, M. Westwood

## Abstract

A method of comparing differences in growth rates of tumours in small groups of animals is described. A common slope can be fitted to the growth curves of a given tumour in a group of isogeneic animals. Differences between growth potentials can be demonstrated by comparing the common slope for a given tumour against that of another tumour (or the same tumour at a later stage of development).

A highly significant difference is shown between the growth potential of an A-strain mammary carcinoma after 7 or after 28 days' growth in isogeneic animals. Since this increase in autonomy is reflected in the tumour's subsequent growth rate in secondary hosts, it is suggested that it involves adaptation of the tumour rather than progressive immunodepression of the primary host.


*Br. J. Cancer* (1974) **29**, 151

### A METHOD OF COMPARING DIFFERENCES IN TUMOUR GROWTH RATES APPLIED TO A STUDY OF THE INCREASING GROWTH CAPACITY OF MOUSE CARCINOMATA

J. A. REES AND M. WESTWOOD

*From the Departments of Surgery and of Extramural Studies, University of Bristol*

Received 5 July 1973. Accepted 13 November 1973

**Summary.—A method of comparing differences in growth rates of tumours in small groups of animals is described. A common slope can be fitted to the growth curves of a given tumour in a group of isogeneic animals. Differences between growth potentials can be demonstrated by comparing the common slope for a given tumour against that of another tumour (or the same tumour at a later stage of development).**

**A highly significant difference is shown between the growth potential of an A-strain mammary carcinoma after 7 or after 28 days' growth in isogeneic animals. Since this increase in autonomy is reflected in the tumour's subsequent growth rate in secondary hosts, it is suggested that it involves adaptation of the tumour rather than progressive immunodepression of the primary host.**

THIS PAPER describes a simple method of analysing differences in tumour growth rates in small groups of animals. An exponential growth model has been shown to give a good fit during the period of maximum tumour growth (Brues, Weinger and Andervont, 1939; Collins, Loeffler and Tivey, 1956; Looney *et al.*, 1973). Using this model, it is possible to fit a common slope to the growth curves of all animals carrying transplants of a given tumour and to compare this with the common slope for the growth of a second tumour in a comparable group of animals. In this way, maximum sensitivity can be obtained to emphasize relatively small differences in tumour sizes and to minimize variability.

This analysis has been applied to the results of experiments investigating the increased aggressiveness of certain mouse tumours during development and in the course of serial transplantation in isogeneic hosts.

Rees and Symes (1971*a*, *b*) demonstrated that on serial transplantation of Bittner virus-induced mammary carcinomata in isogeneic A-strain mice, the tumours showed increasing autonomy but there was no associated reduction in the cellular immune responsiveness of the hosts. Further experiments (Rees and Symes, 1973) demonstrated that 3-methylcholanthrene-induced mouse sarcomata showed a similar increase in autonomy on passage in CBA/H-T6 mice.

It was suggested that this increase in growth capacity was associated with adaptation of the tumour (either by selection of cells or progressive coating with enhancing antibody) rather than immune depression of the hosts. This paper investigates whether changes in a tumour on prolonged growth in its first host would be reflected in its growth potential in secondary hosts.

### MATERIALS AND METHODS

*Animals and tumours.*—Young adult A-strain mice of both sexes, maintained by strict brother and sister mating, have been used throughout. Four mammary carcinomata $B_{22-25}$ which arose in females of the breeding colony were separately passaged subcutaneously through a series of A-strain hosts. The size of tumour transplanted was such that it has a mean diameter of 2 mm, measured externally immediately following transplantation. The transplants were not reduced to cell suspensions and administered as a given number of viable cells since the enzymic degradation necessary to produce a suspension from this type of carcinoma might itself produce erroneous behavioural changes in the tumour by affecting surface antigens or antibody/antigen complexes.

Tumour measurements were made with calipers across the major and minor axes, 3 times a week.

*Experimental plan.*—Tumours $B_{22-25}$ were each transplanted from the A-strain autochthonous host to 6 isogeneic mice—the primary hosts. After 7, 14, 28 or 42 days' growth, the tumours were measured and one was excised, subdivided and passaged (as a standard size transplant) into 6 to 8 A-strain secondary hosts. Tumour growth in these secondary hosts was followed.

*Analysis of tumour growth.*—(1) The method of analysing and comparing the growth characteristics of the tumour is explained in terms of the growth in 3 secondary hosts of samples of tumour $B_{22}$ which had already grown for 7 days in the primary host.

The standard tumour transplants had a mean diameter of 2 mm measured externally immediately following transplantation. Fig. 1 shows the set of points used to fit linear regression lines of log mean diameter on growth time. Tumour sizes exceeding 18 mm were omitted, this being the upper limit of the exponential growth phase for tumours $B_{22-25}$ (a limit probably determined by mechanical factors such as blood supply).

The lines were not constrained to pass through the tumour size of 2 mm mean diameter on Day 0 since many tumours did not start growing appreciably until many days after transplantation.

The coefficients of correlation between log mean tumour diameter and growth time were calculated using these logarithmic values. Fig. 2 shows the fitted lines for all 6 mice with tumours $B_{22}$ (for 7 days' growth in primary host) and $B_{22}$ (28 days' growth in primary host). The slopes of the regression lines for individual mice bearing transplants of $B_{22}$ (7 day) appear very similar to one another, and obviously different from those of $B_{22}$ (28 day). A common slope for the regression lines for individual mice bearing transplants

FIG. 1.—The growth of tumour $B_{22}$ in the 3 example secondary hosts. Fitted regression lines of log mean diameter on growth time.

FIG. 2.—Fitted regression lines for the growth of tumour $B_{22}$ in secondary hosts, after 7 days or 28 days in the primary host.

of a given tumour can be justified by an analysis of variance for all the points for all mice bearing transplants from one tumour. A pooled estimate for this slope can then be calculated (Armitage, 197).

Comparison of growth characteristics can then be made between 2 of the tumour groups to be compared, *e.g.* $B_{22}$ (7 day) and $B_{22}$ (28 day) by comparing the common slope for each group. An analysis of variance for the total variation within all the mice bearing all the tumours to be compared showed:

| Source of variation | Sum of squares | Degrees of freedom | Estimate of variance | F ratio |
|---|---|---|---|---|
| Common slope | 5·424 | 1 | | |
| Difference between slopes | 0·471 | 13 | 0·036 | 5·1 |
| Residual about separate lines | 0·243 | 36 | 0·007 | |
| Total within mice | 6·137 | 50 | | |

The F ratio is significant at 0·5 per cent, showing a highly significant difference between slopes. As each group, *e.g.* $B_{22}$ (7 day) and $B_{22}$ (28 day) taken individually had no significant difference of slopes within them, then the slopes of $B_{22}$, (7 day) with pooled estimate of slope 0·0311, is significantly less than the slopes of $B_{22}$ (28 day) with pooled estimate of slope 0·0554. A computer programme can be written to facilitate these calculations.

(2) A further comparison between the growth curves was made using the time intercepts, *i.e.* the time after transplantation that the fitted line cuts a fixed tumour size, taken in this case as 2 mm mean diameter. As the distribution of these intercepts is not known but appeared considerably skewed, the comparison between different cases was carried out by the non-parametric Mann–Whitney U test.

(3) The survival times of the secondary hosts carrying these tumours were noted.

TABLE I.—*Summary of Analysis of Growth of Tumours $B_{22-25}$*

These A-strain mammary carcinomata were transplanted subcutaneously to isogeneic hosts

| | | | | | | Analysis of variance | | | | | |
|---|---|---|---|---|---|---|---|---|---|---|---|
| | | | | | | Difference between slopes within a given group | | | Difference between slopes within a given tumour | | |
| Tumour number | Size in autochthonous host (mm) | Time in primary host ("Group") (days) | Size at time of transplantation* (mm) | Number of secondary hosts | Pooled estimate of slope | F ratio | Degrees of freedom | Significance at 5% | F ratio | Degrees of freedom | Significance |
| 22 | 14·5 | 7 | 5 | 6 | 0·0311 | 1·49 | (5; 22) | NS | 5·37 | (13; 36) | Sig. at 0·%1 |
| | | 28 | 19 | 8 | 0·0554 | 1·05 | (7; 14) | NS | | | |
| | | | | | | | | Sig. at 5% | | | |
| | | 7 | 4·5 | 6 | 0·0259 | 2·91 | (5; 17) | NS 2·5%, | | | |
| 23 | 11·5 | 14 | 12·5 | 6 | 0·0431 | < 1 | (5; 10) | NS | 3·66 | (19; 34) | Sig. at 0·5% |
| | | 28 | 14 | 6 | 0·0444 | < 1 | (5; 7) | NS | | | |
| 24 | 20 | 7 | 5 | 6 | 0·0391 | < 1 | (5; 11) | NS | 1·36 | (11; 23) | NS at 5% |
| | | 28 | 7 | 6 | 0·0363 | 1·97 | (5; 12) | NS | | | |
| | | 14 | 9 | 6 | 0·0392 | 2·25 | (5; 16) | NS | | | |
| 25 | 14 | 28 | 18·5 | 6 | 0·0549 | 2·07 | (5; 14) | NS | 3·49 | (17; 40) | Sig. at 0·1% |
| | | 42 | 27·5 | 6 | 0·0643 | 1·26 | (5; 10) | NS | | | |

* This tumour was subdivided into " standard size " pieces before transplantation to secondary host.

### RESULTS

### *Tumour growth rates*

The growth of tumours $B_{22-25}$ was studied in 62 mice; of these 44 gave a better fit using an exponential rather than a linear growth model. Only 5 of 62 tumours gave coefficients of correlation less than 0·95 using the exponential model for their growth. The lowest value was 0·927. There was, therefore, good overall support for the use of the exponential model. Table I shows the summary of the analysis of growth of tumours $B_{22-25}$. Tumours 22, 23, 25 showed very high significance in the difference between the pooled slopes of the groups and in only one of these groups was there a significant difference between the slopes within a group. There was therefore a significant difference between the growth rates of the 7 day, 14 day or 28 day groups, for any given tumour. In each case, the tumour transplants which had been longest in the primary hosts grew very much faster than the transplants of the same original tumour, which had been in the primary hosts for a shorter time.

### *Time intercepts*

The time intercepts of the growth curves were compared, *i.e.* the time after transplantation that the fitted line cut a fixed tumour size (taken as 2 mm mean diameter). This gives the time at which the transplant began to grow.

The results are summarized in Table II. The following cases were shown to be significantly different: tumour 22, the 7 day against 28 day group; tumour 23, 7 and 21 day groups against 28; tumour 24, 7 day against 28 day.

In each case, the tumours which had been longest in the primary hosts had lower intercepts, *i.e.* started growth earlier.

### *Survival times*

The survival times of the secondary hosts carrying $B_{22-25}$ are shown in Table

TABLE II.—*Time Interval between Transplantation to Secondary Hosts and Commencement of Tumour Growth*

| Tumour number | Days in primary hosts | Mean no. of days ±s.d. | Significance in Mann-Whitney test |
|---|---|---|---|
| B22 | 7 | 14·3±5·8 | $P<0·05$ |
| | 28 | 10·7±2·4 | |
| B23 | 7 | 10·1±4·9 | 7 *v.* 28 day $P<0·05$ |
| | 21 | 11·4±4·5 | 21 *v.* 28 day $P<0·025$ |
| | 28 | 6·5±1·1 | |
| B24 | 7 | 18·1±8·7 | $P<0·01$ |
| | 28 | 6·7±5·5 | |
| B25 | 14 | 8·2±7·7 | 14 *v.* 42 NS |
| | 28 | 6·3±2·7 | 28 *v.* 42 NS |
| | 42 | 6·1±2·6 | |

TABLE III.—*Survival Times of Secondary Hosts Carrying Tumours $B_{22-25}$*

| Tumour number | Days in primary host | Mean survival times in days ±s.d. | Significance in Mann-Whitney test |
|---|---|---|---|
| B22 | 7 | 77·2±7·1 | $P<0·01$ |
| | 28 | 49·3±6·9 | |
| B23 | 7 | 73·6±8·5 | 7 *v.* 28 day $P<0·05$ |
| | 21 | 63·6±22·1 | 21 *v.* 28 day NS |
| | 28 | 51·1±18·5 | |
| B24 | 7 | 63·8±11·6 | NS |
| | 28 | 53·0±16·6 | |
| B25 | 14 | 51·3±11·7 | 14 *v.* 42 day $P<0·05$ |
| | 28 | 59·3±15·9 | 28 *v.* 42 day $P<0·05$ |
| | 42 | 36·0±5·4 | |

III. In the cases of 22, 23 and 25, the animals carrying the tumours which had been longest in the primary hosts, died significantly sooner.

### DISCUSSION

Previous studies of the cell population kinetics of frequently passaged tumours have been reviewed (Denekamp, 1970; Steel *et al.*, 1971). In particular Looney *et al.* (1973), comparing various models, found that an exponential model most accurately described the growth of rat hepatomata. Exponential growth patterns were applied to mouse mammary tumours by Cheshire (1970) but the slope of each line was drawn on the basis of best fit by eye.

In this paper, the exponential growth of the tumour is described in terms of log mean diameter (proportional to log volume and thus to log cell number). It was shown that a given tumour had a common growth rate in all the isogeneic animals to which it was transplanted, *i.e.* the growth pattern was so characteristic as to be independent of the individual animal. Having established this, then the differences between the growth potential of a tumour at various stages of development was outstanding. A highly significant difference exists between its growth potential after 7 or 28 days' growth in the primary host, which must reflect a difference in the actual doubling time of the cells.

Analysis of the time intercepts showed that in 3 of the 4 tumours the transplants which had been longest in the primary hosts began their growth earlier in the secondary hosts. Finally, the analysis of the survival times of the tumour bearers gave additional support for the idea that the tumours grown longest in the first hosts had become more aggressive.

In previous experiments (Rees and Symes, 1971*a*) it was demonstrated that during serial transplantation in isogeneic animals the A-strain mammary carcinomata showed increased autonomy. This was judged either by the decreasing host lymphoid hyperplasia they evoked or their decreased killing time as passaging continued, but in general no reduction was found in the ability of spleen cells from hosts bearing passages of the same tumour to induce graft-versus-host reactions in $F_1$ hybrid mice. Results of the present experiments suggest that there is increasing tumour autonomy on prolonged growth in one host, and since this is reflected in the tumours' subsequent growth rate in secondary hosts, this must involve adaptation of the tumour rather than progressive immunodepression of the primary host. The way this functions could be by selection of the fastest dividing cells, the least antigenic cells, or a process of progressive coating of the antigeneic determinants by antibody, or by free antigen/antibody complexes. This finding that the growth characteristics of tumours can alter so markedly during development suggests that in experimental systems using second generation tumours the period of growth in the primary tumour should be kept constant.

We should like to thank Dr M. Symes for discussion, and Miss B. Hall and Miss T. Lai for their technical assistance in this project.

This work was supported by a generous grant from the Cancer Research Campaign.

### REFERENCES

ARMITAGE, P. (1971) *Statistical Methods in Medical Research.* Oxford: Blackwell Scientific Publications. p. 150.

BRUES, A. M., WEINGER, A. E. & ANDERVONT, W. B. (1939) Relation between Latent Period and Growth Rate in Chemically Induced Tumours. *Proc. Soc. exp. Biol. Med.*, **43**, 374.

CHESHIRE, P. J. (1970) The Effect of Multiple Tumours on Mammary Tumour Growth Rates in the $C_2H$ Mouse. *Br. J. Cancer*, **24**, 542.

COLLINS, V. P., LOEFFLER, R. K. & TIVEY, H. (1956) Observations on Growth Rates of Human Tumors. *Am. J. Roentg.*, **76**, 988.

DENEKAMP, J. (1970) Cellular Proliferation Kinetics of Animal Tumors. *Cancer Res.*, **30**, 393.

LOONEY, W. B., MAYO, A. A., ALLEN, P. M., MORROW, J. Y. & MORRIS, H. P. (1973) A Mathematical Evaluation of Tumour Growth Curves in Rapid, Intermediate and Slow Growing Rat Hepatomata. *Br. J. Cancer*, **27**, 341.

Rees, J. A. & Symes, M. O. (1971*a*) Observations on the Increasing Malignancy of Tumours on Prolonged Growth. The Influence of Immunological Changes in the Host. *Br. J. Cancer*, **25**, 121.

Rees, J. A. & Symes, M. O. (1971*b*) Further Observations on Whether Host Immunodepression is Associated with Tumour Growth in Mice. *Br. J. Cancer*, **25**, 501.

Rees, J. A. & Symes, M. O. (1973) Immunodepression as a Factor during 3-methylcholanthrene Carcinogenesis and Subsequent Tumor Growth in Mice. *Int. J. Cancer*, **11**, 202.

Steel, G. G., Adams, K., Hodgett, J. & Janik, P. (1971) Cell Population Kinetics of a Spontaneous Rat Tumour during Serial Transplantation. *Br. J. Cancer*, **25**, 802.